# After the Resistance: The Alamo Today

**DOI:** 10.3201/eid2007.AC2007

**Published:** 2014-07

**Authors:** Byron Breedlove, Murray L. Cohen

**Affiliations:** Centers for Disease Control and Prevention, Atlanta, Georgia, USA; US Public Health Service (retired), Fort Worth, Texas, USA

**Keywords:** art science connection, emerging infectious diseases, art and medicine, Eileen Pestorius, The Alamo, After the Resistance: the Alamo Today, Texas revolution, antimicrobial resistance, antibiotics, bacteria, Clostridium difficile, methicillin-resistant Staphylococcus aureus, pathogens, microbes, penicillin, war, Sir Alexander Fleming, about the cover

**Figure:**
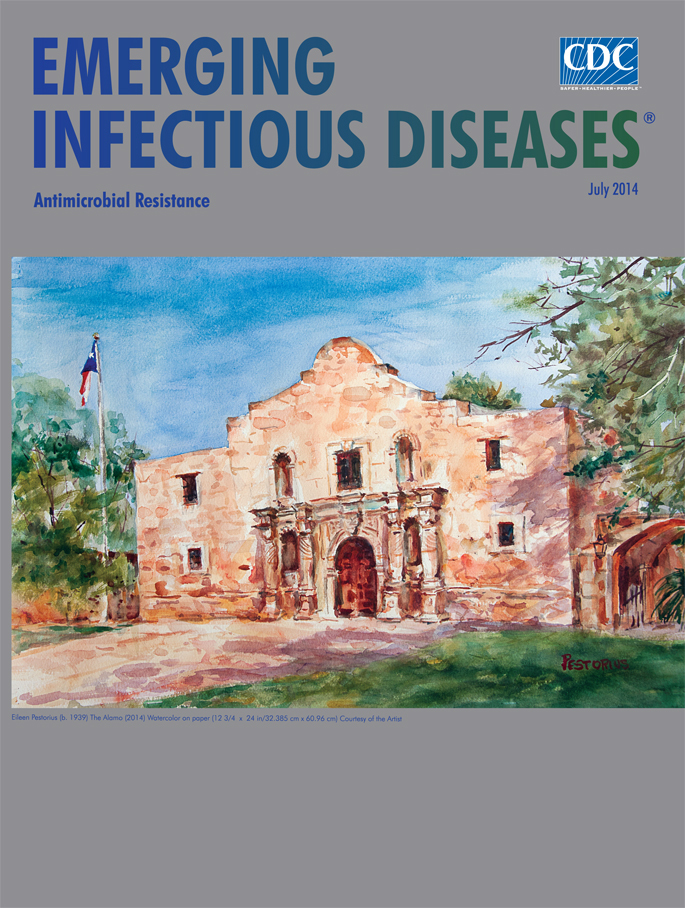
**Eileen Pestorius (b. 1939) The Alamo (2014) Watercolor on paper (12.75 in x 24 in/32.385 cm x 60.96 cm)** Courtesy of the Artist

San Antonio de Valero is a former Roman Catholic mission and fortress where the Battle of the Alamo occurred from February 23 until March 6, 1836. Now known simply as the Alamo, this compound has been damaged, ransacked, and renovated many times before work on its present configuration as a museum in downtown San Antonio, Texas, USA, was completed in 1968.

Texas artist Eileen Pestorius used watercolors to capture the setting on a still, spring day. In her painting, trees and grass frame the mission; a flagstone pathway leads to the entrance, and thin clouds swirl overhead in a hazy blue sky. A Texas flag hangs limply on this calm day. The stonework looks weathered, even warm in the bright sunlight; a large wooden door flanked by pairs of columns invites exploration, as does an archway that juts from the right and seems to lead to a garden.

Although located in a bustling urban setting, the modern day Alamo seems a tranquil, restive place suited for relaxation or contemplation. It takes some effort to associate the Alamo that Pestorius has rendered with the garrison known as an iconic symbol of heroic fighting and resistance.

The story is well known. During the 1836 battle, a Mexican force numbering in the thousands and led by General Antonio Lopez de Santa Anna besieged the Alamo. The vastly outnumbered defenders—200 men, including the frontiersman Davy Crockett, commanded by Colonel James Bowie and Lieutenant Colonel William Travis—valiantly held the compound for 13 days before the Mexicans breached the mission, killing nearly everyone. On April 21, 1836, when the outnumbered Texan militia commanded by General Sam Houston defeated Santa Anna’s troops at San Jacinto, the rallying cry was “Remember the Alamo!” Houston’s surprise attack proved such a tactical advantage that he routed the larger force in just 18 minutes, effectively ending the Texas Revolution and leading to treaties that established the independent Republic of Texas.

Our often-used metaphor of humans waging “war” on infectious diseases and on the microbes that cause them invites comparison with the Battle of the Alamo. Through various antibiotics, we reduced the number of deaths caused by infections and, bolstered by successes, assumed we would win this war.

In reality, we live in a microbial world where we are the invaders. The Board on Global Health and Institute of Medicine noted that “On reflection, perhaps it would be wise to reconsider the frequently used metaphor of humans being ‘at war with microbes.’ It is absurd to believe that we could ever claim victory in a war against organisms that outnumber us by a factor of 10^22^, that outweigh us by a factor of 10^8^, that have existed for 1,000 times longer than our species, and that can undergo as many as 500,000 generations during 1 of our generations.”

The Centers for Disease Control and Prevention estimates that in 2013, antibiotic resistance threats caused more that 2 million illnesses and 23,000 deaths in the United States and that in 2011, those threats were responsible for an estimated $20 million in excess health care costs, 8 million additional hospital days, and $35 million in societal costs. Many factors, including overuse and misuse of antibiotics, global climate change, human encroachment into more remote, less hospitable places, modern factory farming and food production practices, and rapid and accessible global mobility have heightened our vulnerability. Since the 1980s, nearly 40 new pathogens have been identified as human disease threats, and 12% of known human pathogens have been classified as either emerging or remerging.

The cycle of many of these emerging microbes can be seen with methicillin-resistant *Staphylococcus aureus* infections moving from being hospital associated to community acquired and now to a ubiquitous supply-chain associated infection. *Clostridium difficile* seems headed on the same path.

Successfully controlling drug-resistant microbes requires not just greater vigilance with our infection control tools, but it also requires developing and deploying creative and aggressive tactics. Changing our tactics against drug-resistance microbes involve keeping pathogens out of our supply chains to schools, hospitals, and workplaces, and keeping sick workers at home. New guidelines from the Society for Healthcare Epidemiology of America, for example, provide recommendations to reduce the role that health care personnel attire plays in the cross-transmission of pathogens.

The scenario of a postantibiotic era of infectious diseases that looks like the preantibiotic era that preceded penicillin and vaccines is an alarming scenario. Sir Alexander Fleming even warned about antibiotic resistance in his 1945 Nobel Prize speech. Now is the time to incorporate new strategies into our battle plans and prepare to fight against overwhelming odds.
